# Plasma-Activated Water Against Carbapenem-Resistant *Klebsiella pneumoniae* and Vancomycin-Resistant *Enterococcus faecalis*

**DOI:** 10.3390/pathogens14050410

**Published:** 2025-04-24

**Authors:** Dragana Vuković, Maja Miletić, Boško Toljić, Nikola Milojević, Olivera Jovanović, Jovana Kuzmanović Pfićer, Nikola Škoro, Nevena Puač

**Affiliations:** 1 Faculty of Medicine, University of Belgrade, Dr Subotića starijeg 8, 11000 Belgrade, Serbia; 2 School of Dental Medicine, University of Belgrade, Dr Subotića starijeg 8, 11000 Belgrade, Serbia; bosko.toljic@stomf.bg.ac.rs (B.T.); nikola.milojevic@stomf.bg.ac.rs (N.M.); jovana.kuzmanovic@stomf.bg.ac.rs (J.K.P.); 3 Institute of Physics, University of Belgrade, Pregrevica 118, 11000 Belgrade, Serbia; olivera@ipb.ac.rs (O.J.); nskoro@ipb.ac.rs (N.Š.); nevena@ipb.ac.rs (N.P.)

**Keywords:** plasma-activated water, antibacterial efficacy, multidrug resistance, *Klebsiella pneumoniae*, Enterococcus faecalis

## Abstract

The scope of the antibacterial effects of plasma-activated water (PAW) is not yet fully comprehended. We investigated the activity of PAW produced by the in-house 3-pin atmospheric pressure plasma jet against carbapenem-resistant *Klebsiella pneumoniae* and vancomycin-resistant *Enterococcus faecalis*, with a focus on PAW’s potential to promote susceptibility to conventional antibiotics in these bacteria. Bacterial inactivation was determined by the colony count after 15 and 60 min PAW treatments. Minimum inhibitory concentrations (MICs) measured following repeated exposures to PAW across multiple generations of bacteria enabled the assessment of changes in susceptibility to antibiotics. The PAW’s efficacy was also analyzed through the detection of intracellular reactive oxygen and nitrogen species in treated bacteria. Time-dependent significant inactivation efficiency against *K. pneumoniae* was observed (log reduction 6.92 ± 0.24 after 60 min exposure), while effects on *E. faecalis* were limited. PAW demonstrated potential to decrease the MICs of crucial antibiotics. Namely, a 50 to 62.5% decrease in the MICs of colistin against *K. pneumoniae* and a 25% reduction in the MICs of vancomycin against enterococci were recorded. We found a significant increase in the superoxide anion concentration in *K. pneumoniae* and *E. faecalis* cells after PAW treatments. This study indicates that PAW’s inactivating efficacy coupled with the capacity for the potentiation of antibiotic effects is a promising combination against multidrug-resistant bacteria.

## 1. Introduction

Antimicrobial resistance (AMR) is one of the most pressing global health threats we face today [1,2]. The resistance of bacteria to antibiotics is of particular importance, as multiple recent surveys and surveillance studies show alarmingly high and still increasing levels of bacterial AMR with devastating consequences [1,2,3,4,5]. It has been shown that bacterial AMR, associated with 4.95 million deaths, was a leading cause of death globally in 2019 [4]. According to the most recent forecasts, further increases in the counts are expected [5]. The estimated numbers of annual deaths attributable to and associated with bacterial AMR in 2050 will be 1.91 million and 8.22 million, respectively.

It is therefore clear that responding to bacterial AMR with novel therapeutics is of paramount importance [2,5,6]. The World Health Organization (WHO) addressed the challenge by classifying the antibiotic-resistant bacterial pathogens into the critical, high, and medium categories of priority [7]. The major purpose of this list of priority pathogens, which was first published in 2017 and updated in 2024, is to guide research and the development of new antibacterial agents for control, prevention, and treatment. While the latest review of antibiotics in clinical and preclinical development reveals some promising candidates, the overall finding is a lack of new antibiotics to effectively combat drug-resistant bacteria recognized as priority pathogens [6,8].

Novel non-traditional agents capable of overcoming multiple drug resistance mechanisms in bacteria offer the potential to tackle bacterial AMR. Cold atmospheric plasma (CAP), a non-thermal ionized gas generated at ambient temperature and pressure, is an innovative technology that has emerged as a highly promising option targeting antibiotic-resistant bacteria. The antibacterial activity of CAP has been shown in multiple studies and is attributed primarily to the mixture of reactive oxygen and nitrogen species (RONS) that are created in the gas phase of the discharge [9,10,11,12,13,14,15,16,17]. During CAP treatments, bacteria can be in direct contact with the plasma gas phase and its rich chemistry containing short- and long-lived RONS, ions, electrons, UV radiation, and metastable and excited species or indirect contact, when CAP is used to treat suspensions containing bacteria or liquid to produce plasma-activated liquid (PAL), including plasma-activated water (PAW) [18,19,20,21,22,23,24,25,26].

CAP is used for treatments of deionized, distilled or tap water, then stored for some period or applied to microbial samples immediately after the CAP treatments. The characteristics of PAW vary depending on the type of CAP source that is used, its electrode geometry, working gas, frequency, the type of the applied voltage (sine, pulsed), and if the discharge is created above the water or in bubbles in the water [27,28,29,30,31,32,33]. The chemistry responsible for bacterial inactivation primarily includes short-lived RONS (like hydroxyl radicals (OH^•^), ozone (O_3_), superoxide (O_2_^−^), peroxynitrite (ONOOH), etc.) and long-lived RONS (nitrate (NO_3_^−^), nitrite (NO_2_^−^), hydrogen peroxide (H_2_O_2_), etc.) [18,34,35,36,37]. In the literature, characteristics of PAW are usually reported by the measured pH, oxidation reduction potential (ORP), conductivity, and, more importantly, by measured concentrations of H_2_O_2_, NO_3_^−^, and NO_2_^−^, which are the main reasons for the bactericidal properties of PAW. The complete understanding of mechanisms leading to bacterial inactivation is still lacking, but it has been shown that the cell membrane and cell wall, intracellular proteins, and DNA are key bacterial structures and components affected by RONS contained in PAW [19,20,21,25].

Many studies have reported PAW’s potential to inactivate bacteria [38,39,40,41,42,43], but also showed high variability in the results obtained due to processing parameters for PAW generation and application as well as bacterial characteristics. We, therefore, aimed to assess the inactivation efficiency of PAW produced by the in-house 3-pin atmospheric pressure plasma jet (3-pin APPJ) against selected model multidrug-resistant (MDR) bacteria, carbapenem-resistant *Klebsiella pneumoniae* and vancomycin-resistant *Enterococcus faecalis.* Both bacteria are among the leading causes of healthcare-associated infections (HAIs) [44,45], and their resistance patterns have been recognized as particularly challenging targets for conventional antimicrobials [1,2,3,7]. Nevertheless, they have not been extensively investigated as specific targets for the antimicrobial activity of PAW, which particularly applies to *K. pneumoniae*. Several previous studies showed CAP and plasma-activated saline treatments to result in enhanced antibiotic activity [46,47,48], but these data for the effects of PAW treatments are lacking. Hence, the second goal of our research was to determine PAW’s potential to promote the effects of antibiotics against selected MDR bacteria. In addition, the PAW’s efficacy was also analyzed through the detection of intracellular RONS in treated bacteria.

## 2. Materials and Methods

### 2.1. Experimental Setup and Plasma Treatment of Water Sample

**Figure 1:**
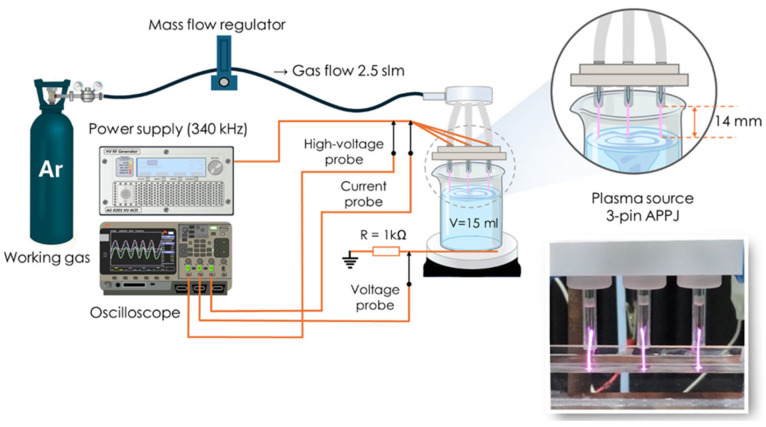
Three-pin atmospheric pressure plasma jet (3-pin APPJ) configuration.

The 3-pin APPJ was used as a plasma source for the production of RONS in a water sample (Figure 1). The system operated with argon (Argon 5.0) as a working gas at a flow rate of 2.5 slm, regulated by a mass flow controller (Mass View MV-304, Bronkhorst High-Tech B.V., Ruurlo, The Netherlands). A detailed description of the 3-pin APPJ has been provided in a previous study [49]. The 3-pin APPJ was constructed by using three syringe needles, functioning as powered electrodes, enclosed within transparent quartz tubes. The needle tips were positioned 5 mm from the endings of the quartz tubes.

The grounded electrode was placed below a treated water sample, which was placed in a plastic Petri dish under the 3-pin APPJ. A copper tape was attached to the bottom of Petri dish and grounded through a 1 kΩ resistor. The distance between the needle tips and the water surface was maintained at 14 mm.

The 3-pin APPJ was powered by a high voltage power supply (AG 0201 HV-ACD RF HV GENERATOR, T&C Power Conversion, Inc., Rochester, NY, USA) operating at a continuous sinusoidal wave signal of 340 kHz. Electrical parameters, including the high voltage applied to the 3-pin APPJ and the current through it, were continuously monitored and controlled to ensure consistent treatment conditions. A high-voltage probe (P6015A, Tektronix, Inc., Beaverton, OR, USA) and a current probe (N2783B, Agilent Technologies, Tokyo, Japan) were used for these measurements. Additionally, the current passing through the grounded line was determined by measuring the voltage drop across the 1 kΩ resistor (by using probe TPP0201, Tektronix, Inc., Beaverton, OR, USA). The discharge power, calculated as described previously [50], was maintained at an average value of 14 W during all treatments.

To create PAW, which was used for all experiments on bacteria, plasma treatments were conducted on 15 mL of deionized water (Milli-Q, pH 7.3) for 10 min. Prior to treatment, 25 mg of ZnO was added to prevent sample acidification caused by plasma exposure [51].

### 2.2. Characterization of Plasma-Activated Water

To investigate the effects of plasma–liquid interaction, after filtering the sample to remove remaining ZnO, the pH value (HI1131 pH electrode, HI5521 controller, Hanna Instruments Inc., Woonsocket, RI, USA) and concentrations of the hydrogen peroxide, nitrate, and nitrite of treated water were determined. All measurements were made in triplicate immediately after the plasma treatment.

The concentrations of three reactive species (H_2_O_2_, NO_3_^−^, and NO_2_^−^), in PAW were determined photometrically by using a UV-VIS spectrophotometer (UV/VIS Lambda 365, PerkinElmer, Inc., Waltham, MA, USA). Prior to the experiments, calibration curves were made for each reactive species. RONS concentrations exceeding the measurement range of the methods were quantified by diluting the samples with deionized Milli-Q water prior to analysis.

The hydrogen peroxide concentration in treated liquid was quantified using the titanium oxysulfate (TiOSO_4_) assay (Titanium(IV) oxysulfate - sulfuric acid solution, Sigma Aldrich Chimie S.a.r.l, St. Quentin Fallavier Cedex, France), which measures the absorbance of yellow colored pertitanic acid formed in the reaction between H_2_O_2_ and titanyl ions in acidic conditions [52]. One volume of reagent per ten volumes of aqueous solution containing H_2_O_2_ was added [52] and the absorption peak value at 407 nm was measured. Calibration curves were prepared by using seven different H_2_O_2_ concentrations (0–50 mg/L) obtained by diluting a 30% stock solution (1.07210.1000 Hydrogen peroxide 30%, Merck KgaA, Darmstadt, Germany).

Nitrate (NO_3_^−^) concentrations were determined with the Spectroquant Nitrate Test (1.09713.0002, Merck KgaA, Darmstadt, Germany), based on the nitration of 2,6-dimethylphenol to 4-nitro-2,6-dimethylphenol with an absorption maximum at λ = 357 nm [33]. A calibration curve (0–110 mg/L) was constructed using dilutions of a nitrate stock solution (200 mg/L NO_3_^−^-N, Merck KgaA, Darmstadt, Germany).

Nitrite (NO_2_^−^) concentrations were measured by using the Spectroquant Nitrite Test (1.14776.0001, Merck KgaA, Darmstadt, Germany), where NO_2_^−^ reacts with Griess reagent in acidic conditions to form a red-violet azo dye (λ = 525 nm) [53]. Calibration was performed using dilutions of a nitrite stock solution (40 mg/L NO_2_^−^-N, Merck KgaA, Darmstadt, Germany) in the range of 0.07–3.28 mg/L.

The pH value of obtained PAW was 6.8 ± 0.1, and concentrations of H_2_O_2_, NO_2_^−^, and NO_3_^−^ were 56 ± 4 ppm, 55 ± 3 ppm, and 74 ± 4 ppm, respectively. All PAW samples used were checked for sterility by inoculation on tryptic soy agar (TSA).

### 2.3. Bacterial Strains and Growth Conditions

The reference carbapenem-resistant *K. pneumoniae* ATCC BAA-2814 and the reference vancomycin-resistant *E. faecalis* ATCC 51299 strains were used in the study. The *K. pneumoniae* ATCC BAA-2814 strain displays resistance to aminoglycosides, fluoroquinolones, and beta-lactams, and is considered a model strain for carbapenem-resistance in enterobacteria, while *E. faecalis* ATCC 51299 is high-level gentamicin and vancomycin resistant.

The master stocks were maintained at −20 °C with 40% of glycerol. The strains were plated on TSA and incubated aerobically for 24 h at 37 °C. A single colony was transferred from the plates into 10 mL of fresh tryptic soy broth (TSB), and following incubation at 37 °C for 16 h, a culture was diluted to obtain a final concentration of 1 × 10^8^ CFU/mL determined by using a McFarland densitometer (DensiCHEK plus, bioMerieux, Marcy-l’Étoile, France).

### 2.4. PAW Treatment and Assessment of Bacterial Inactivation

For each bacterial species tested, an aliquot of 100 µL of the 1 × 10^8^ CFU/mL bacterial suspension was added to 900 µL of PAW in microtubes and homogenized. The samples containing bacterial load and PAW were held at room temperature for two exposure times (15 min and 60 min) before they were used for further testing. In all experiments, we used bacterial suspensions (100 µL) added to saline (900 µL) as control untreated samples.

The colony forming unit (CFU) assay was used for the assessment of bacterial inactivation by PAW treatment. After the treatments, bacterial suspensions were serially diluted 10-fold in saline. One hundred microliters of each dilution were evenly spread using a bent inoculation loop as a cell spreader on Luria Bertani (LB) agar (for *K. pneumoniae*) or TSA (for *E. faecalis*) plates. After overnight incubation at 37 °C, colonies were counted to determine the number of viable bacterial cells (CFU/mL) and log reduction was calculated using the following equation: Log10Reduction = log10 (CFUcontrol/CFUtreated). All experiments were carried out in triplicate and repeated three times.

### 2.5. Determination of Minimum Inhibitory Concentrations (MICs)

Bacteria were grown and then treated with PAW as described above for the assessment of bacterial inactivation. Following the treatments, an aliquot of 100 µL of bacterial suspensions was used to subculture the surviving bacteria and inoculated onto LB or TSA. After overnight incubation at 37 °C, the grown colonies were collected for the preparation of a 1 × 10^8^ CFU/mL next-generation bacterial suspension, which was then exposed to PAW under the aforementioned conditions. The initial culture before the first PAW treatment was considered as generation 0, followed by generation 1 after the first plasma treatment. In total, the procedure was repeated four times, so that four generations of bacteria were treated with PAW and examined for their susceptibility to antibiotics after the first and fourth exposure to PAW.

The procedure for the determination of MICs was identical in both generations, 1 and 4, of the bacterial strains tested as well as for the untreated bacteria, which served as the control. The 1 × 10^8^ CFU/mL suspensions were prepared from untreated or PAW-treated bacteria, and an aliquot of 100 µL of the suspensions was plated on Mueller Hinton (MH) agar plates. The Liofilchem^®^ MIC Test Strips (Liofilchem, Roseto degli Abruzzi, Italy) were used as a quantitative assay for determining the MICs of selected antibiotics for the bacteria tested. The following MIC test strips were used (the antibiotic gradient scale is given in parentheses): for *K. pneumoniae*, ceftazidime (0.016–256 µg/mL), cefotaxime (0.016–256 µg/mL), gentamicin (0.016–256 µg/mL), levofloxacin (0.002–32 µg/mL), imipenem (0.002–32 µg/mL), and ertapenem (0.002–32 µg/mL), and for *E. faecalis*, vancomycin (0.016–256 µg/mL), teicoplanin (0.016–256 µg/mL), and imipenem (0.002–32 µg/mL). After 24 h incubation, the MIC values were read directly from the scale, at the point where the edge of the inhibition ellipse intersects with the MIC test strip. In addition, the automated Vitek^®^ 2 (bioMerieux, Marcy-l’Étoile, France) system was used for measuring the MICs of colistin against *K. pneumoniae.* The experiments were performed in triplicate and repeated two times.

### 2.6. Detection of Intracellular RONS Generated by the PAW Treatment

Two fluorescent probes, MitoSox™ Red (Thermo Fisher, Waltham, MA, USA) mitochondrial superoxide indicator and the OxiSelect™ in vitro ROS/RNS assay kit (Cell Bio Labs, San Diego, CA, USA) were used to detect intracellular reactive species generated by the PAW treatments applied as previously described.

The detection of superoxide anion (O_2_^•−^) by using MitoSOX™ Red reagent was performed in accordance with the procedure described by Guo et al. [46]; namely, the indicator was incubated with each bacterial sample at a final concentration of 5 µM for 30 min at 37 °C. To detect highly reactive oxygen/nitrogen species, the OxiSelect™ probe, prepared according to the manufacturer’s recommendations, was incubated with bacterial strains for 40 min in the dark at room temperature. Bacteria were then collected by centrifugation, washed three times in saline and 200 µL per treated and untreated bacterial suspensions were transferred to a black 96-well microtiter plate (ThermoNunc 96F, Thermo Scientific, Roskilde, Denmark). Fluorescence measurements were performed using a microplate reader Fluoroskan (Thermo Fisher, Waltham, MA, USA), with an excitation wavelength of 515 nm and an emission wavelength of 578 nm for the MitoSOX™ probe, while the excitation and emission wavelengths of 485 nm and 538 nm, respectively, were used for dichlorodihydrofluorescein (DCF) in the OxiSelect™ probe.

All fluorescence intensities were normalized to the baseline value and results are presented as means ± standard deviation. Untreated bacterial suspensions in saline were used as controls. Each sample was read in three wells and replicated twice.

### 2.7. Statistical Analyses

Data were analyzed in the IBM SPSS Statistics for Windows, version 22.0 (IBM, Armonk, NY, USA). Numerical data were described by using average and standard deviation. The Kolgomor–Smirnov test was used to test the distribution of normality of the data. For parametric data, a one-way ANOVA was used to compare more than two groups, and Bonferroni was used for post hoc analyses. Nonparametric data for comparing the two groups were processed with the Mann–Whitney U test. Statistical significance was set at 0.05.

## 3. Results

### 3.1. Inactivation Efficacy of PAW

**Figure 2:**
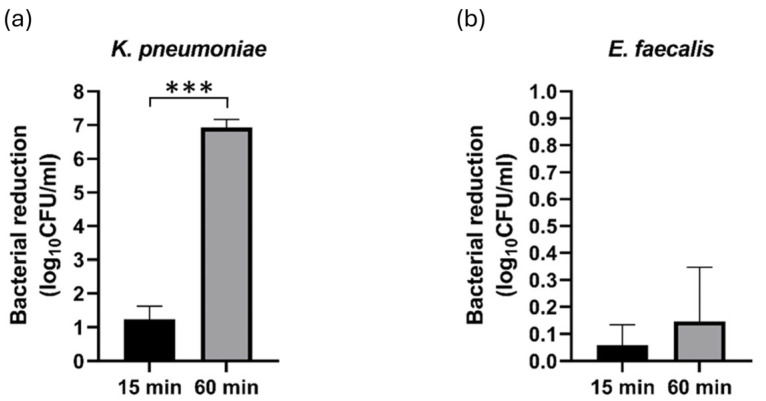
Growth reduction after plasma-activated water (PAW) treatments for 15 and 60 min against (**a**) *K. pneumoniae* and (**b**) *E. faecalis*. Data are representative of three independent experiments carried out in triplicates. Statistical significance is denoted as *** *p* < 0.001.

The bacterial species included in the study exhibited different behaviors in the colony count trials, as is depicted in Figure 2. The most notable reduction of bacterial CFU count was observed after a 60 min PAW exposure of the *K. pneumoniae* strain (log reduction = 6.92 ± 0.24), while the log reduction after a 15 min treatment was 1.23 ± 0.39 (*p* < 0.001) (Figure 2a). In contrast, the overall antimicrobial effects of PAW against the *E. faecalis* strain were limited (Figure 2b), regardless of the duration of treatment (*p* = 0.073).

### 3.2. Effects of PAW on Susceptibility to Antibiotics

**Figure 3:**
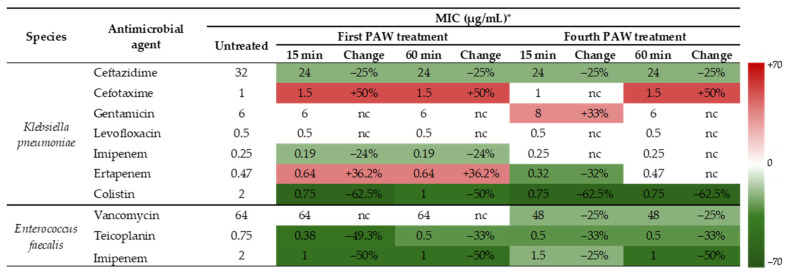
Heat-map plot of minimum inhibitory concentrations (MICs) of selected antibiotics against *K. pneumoniae* ATCC BAA-2814 and *E. faecalis* ATCC 51299 following exposure to PAW for 15 and 60 min across four generations. The change is given with respect to the untreated data. * Data are representative of two independent experiments carried out in triplicates. Nc, not changed.

By exposing bacteria to repeated PAW treatments across multiple generations, we aimed to create conditions similar to those encountered during real-world PAW application in parallel with the continuous multiplication of bacteria. The summarized readings of MIC values are presented in Figure 3. The European Committee on Antimicrobial Susceptibility Testing (EUCAST) breakpoints for categorizing *K. pneumoniae* and *E. faecalis* strains as susceptible (S and I) and resistant (R) to the antibiotics were not applied. Only the differences in MIC values of antibiotics included in the testing against untreated and PAW-treated bacterial samples were of interest as indicators of increase or decrease in susceptibility.

The effects exhibited by the PAW treatments on *K. pneumoniae* susceptibility to antibiotics varied from antibiotic to antibiotic, even within the same group. This was shown for the third-generation cephalosporins and carbapenems tested. While the susceptibilities to cefotaxime either decreased or remained the same as for the controls, MICs of ceftazidime were reduced by 25% under all treatment conditions compared to the untreated control. The effects of PAW treatments on susceptibility to ertapenem were inconsistent, while MICs of imipenem decreased after the initial PAW treatments (0.19 µg/mL). Sensitivities to an aminoglycoside representative, gentamicin, and a fluoroquinolone, levofloxacin, were not affected by PAW treatments. On the other hand, the effects of PAW treatment on *K. pneumoniae* susceptibility to colistin were positive. All modalities of PAW treatments led to a significant decrease in the MIC values of colistin, in comparison to the MIC of 2 µg/mL in untreated control.

The MIC of vancomycin measured against the untreated *E. faecalis* was 64 µg/mL, and remained unchanged after the first treatment with PAW. However, after the fourth treatment, the MIC decreased to 48 µg/mL for both 15 and 60 min exposures, which is a noteworthy result. The *E. faecalis* susceptibilities to teicoplanin and imipenem were also positively affected by the PAW treatments, regardless of the conditions applied (Figure 3).

### 3.3. Assessment of Intracellular RONS Generated by the PAW Treatment

**Figure 4:**
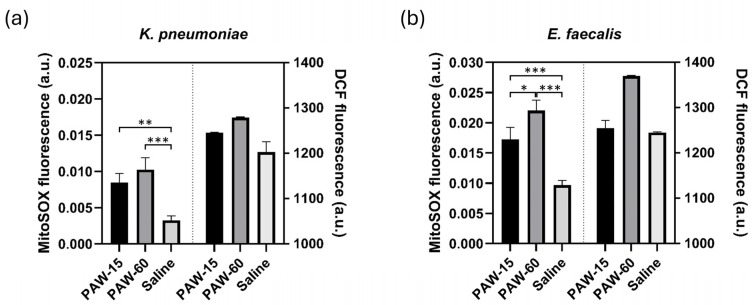
Superoxide anion radical •O_2_^−^ levels detected by MitoSOX™ and hydrogen peroxide (H_2_O_2_), peroxyl radical (ROO·), nitric oxide (NO), and peroxynitrite anion (ONOO-) levels detected by OxiSelect™ assay (DCF) in (**a**) *K. pneumoniae* ATCC BAA-2814 and (**b**) *E. faecalis* ATCC 51299 cells following exposure to plasma-activated water (PAW) for 15 and 60 min. Untreated bacterial cells suspended in saline served as control. Data are representative of two independent experiments. Statistical significance is denoted as * *p* < 0.05; ** *p* < 0.01; and *** *p* < 0.001.

Gram-negative and Gram-positive bacteria differed in the intracellular levels of the reactive species detected by the assays employed (Figure 4). For the *K. pneumoniae* strain (Figure 4a), the fluorescence intensities of the MitoSOX™ probe increased by 2.6-fold (*p* = 0.002) and 3.1-fold (*p* < 0.001) compared to control, after 15 and 60 min of PAW exposure, respectively. In *E. faecalis*, the intracellular concentration of •O_2_^−^ was positively correlated with the time of exposure (Figure 4b). The increase in fluorescence achieved with 60 min exposure was significantly higher than that measured after 15 min treatments (*p* = 0.015). As compared to the control, the highest fluorescence levels were recorded in *E. faecalis* cells after PAW treatment for 60 min (*p* < 0.001). As far as long-lived RONS detected with the OxiSelect™ probe are concerned, the fluorescence increases in both *K. pneumoniae* (Figure 4a) and *E. faecalis* cells (Figure 4b) remained below the level of significance, and no correlation with the treatment time was observed.

## 4. Discussion

PAW as an antimicrobial agent is considered environmentally friendly and cost-effective, and its important advantages over direct CAP treatment are convenience and safety in use [35,54,55]. Accordingly, the scope of promising implementations in medicine, agriculture and environmental applications is growing [15,36,56,57]. The medical and dental fields offer broad opportunities for the clinical implementation of PAW as an alternative antimicrobial strategy, ranging from the decontamination of medical and dental devices to in vivo disinfection and infection control [39,55,58,59]. While PAW is already being described as a novel disinfectant, a validated application as an antimicrobial technology has not been realized so far.

The reference strains used in our study were selected for their multiple drug resistance determinants, primarily resistance to carbapenems in *K. pneumoniae* and resistance to vancomycin in *E. faecalis*. Both patterns of resistance have been recognized as major health threats [2,4,5,7], and are therefore adequate targets for testing PAW as a tool in combating MDR bacteria.

*K. pneumoniae* is a Gram-negative opportunistic pathogen that can cause a variety of HAIs, including pneumonia, sepsis, urinary tract infections, and wound infections [44]. Infections caused by carbapenem-resistant strains are particularly concerning due to their high mortality rates, especially among immunocompromised patients and patients in critical conditions [60]. Therefore, there is a growing necessity for alternative antimicrobial strategies as the effectiveness of last-resort antibiotics against *K. pneumoniae* continues to decline. It is also noteworthy that the species is capable of persisting on various surfaces, and is difficult to eradicate due to resistance to extreme conditions and antimicrobial agents [44]. *E. faecalis* is another example of an opportunistic pathogen, which successfully adapts to and thrives in harsh environments. This Gram-positive bacterium has also been recognized as a major causative agent of HAIs [45]. The species is characterized both by intrinsic resistance to a variety of antibiotics and by its ability to easily acquire resistance to further antibiotics, such as high-level aminoglycoside resistance, and vancomycin resistance [61].

The PAW tested showed inactivation efficiency against Gram-negative *K. pneumoniae*, which increased with contact times to the samples. The effects on Gram-positive *E. faecalis*, on the other hand, were limited regardless of the exposure time. It has already been shown that Gram-positive bacteria display elevated resistance to plasma treatments either when the bacterial load is in contact with plasma (gas phase or as a suspension) [62,63,64] or in case of treatments with PAW [41,65,66]. On the other hand, Nicol et al. [67] showed that both Gram-positive and Gram-negative bacteria were similarly affected by the CAP treatments, while Tipa et al. [68] demonstrated a higher efficacy of CAP against Gram-positive than against Gram-negative bacteria. Increased protection against both CAP and PAW treatments in Gram-positive bacteria has been primarily attributed to the robust cell wall, which is characterized by a thick layer of peptidoglycan reaching a size of between 30 and 100 nm [69]. Given that we used a vancomycin-resistant *E. faecalis* strain, it is noteworthy that reduced susceptibility to plasma treatment was reported in strains of enterococci resistant to vancomycin in comparison to the vancomycin-susceptible counterparts [70]. However, the causality of this finding was not demonstrated. The recent study by Droste et al. [42] investigated whether Gram-positive bacteria are generally robust or truly tolerant to PAW treatment, and established that the complete inactivation of multiple species of Gram-positive bacteria was achievable with longer contact times and a higher concentration of RONS in applied PAW. These mixed findings suggest that different susceptibility to PAW treatments between Gram-positive and Gram-negative bacteria is determined by multiple factors encompassing CAP generation and PAW activation settings, operational parameters of the treatments applied to the bacterial samples, and the complex structure of the cell wall and its appendices in bacteria as well as antioxidant mechanisms in different bacterial species and even strains. Given that this list is not exhaustive, there is at least one direction for our further work based on the results obtained so far. The stark difference between the effects exerted on Gram-negative *K. pneumoniae* viability and the effects on the survival of Gram-positive *E. faecalis* observed in our study indicates a need for the further optimization of the PAW’s antibacterial activity through the optimization of water activation settings and PAW treatment parameters. We have already shown that the effectiveness of PAW in inactivating bacteria improves by increasing the concentration of RONS (by decreasing the water volume used for PAW preparation) and extending the incubation time of the bacteria in PAW [71]. The inconsistencies in the results presented in the literature, including ours, also show that further studies of bacterial inactivation achieved by different PAWs generated by different systems are needed.

The influence of treatment with CAP and, in particular, PALs on the susceptibility of pathogenic bacteria to antibiotics has not been extensively studied. Hoon Park et al. demonstrated that argon plasma, generated by a dielectric barrier discharge plasma device, increased susceptibility to β-lactams in *Staphylococcus aureus* strains, including methicillin-resistant *S. aureus* (MRSA) [47]. In a more recent study, researchers reported that CAP pretreatment increased the sensitivity of both planktonic and biofilm cells of *Pseudomonas aeruginosa* to several antibiotics [48]. However, it has also been shown that CAP pretreatment could lead to increased resistance to antibiotics [72], probably through the induction of mutations and adaptive changes. In the case of PALs, a study showed that treatment with plasma-activated saline positively affected the sensitivity of MRSA to five different classes of antibiotics [46]. As far as the effects of PAW treatments on bacterial susceptibility are concerned, the data are lacking, while carbapenem-resistant *K. pneumoniae* and vancomycin-resistant enterococcus have not been investigated in this context so far.

We showed that the effects of PAW on bacterial susceptibility to conventional antibiotics are similar to those achieved with CAP and other PALs [46,47,48,72]. Namely, the effectiveness of PAW treatments in terms of enhancing antibiotic activity against the bacteria tested was not fully consistent, but nevertheless relevant both in Gram-negative and in Gram-positive bacteria. Effects on *K. pneumoniae* susceptibility to third-generation cephalosporins and carbapenems were of interest as enterobacteria resistant to these groups of antibiotics pose the highest estimated burden among all MDR Gram-negative bacteria [4], and are included in the critical priority category in the latest WHO priority pathogen list [7]. With the exception of the MICs of the third-generation cephalosporin ceftazidime that decreased following PAW exposure and, thus, were positively affected, the results obtained with another cephalosporin, cefotaxim, as well as with carbapenems were inconsistent. Nonetheless, the result of particular importance is the decrease in the MICs of colistin as treatment with this antibiotic is considered a salvage therapy for patients infected with drug-resistant *K. pneumoniae* [73]. The MICs of colistin were positively affected by the PAW treatments for both 15 and 60 min, with the decrease ranging from 50 to 62.5%. In contrast to the mixed results obtained for *K. pneumoniae*, PAW positively changed the activity of all antibiotics tested against *E. faecalis.* The reduction in the MIC of imipenem ranged from 25% to 50%, with two-fold reduction achieved in generation 1 regardless of the treatment time. The effects on MICs of glycopeptide antibiotics, which are critically important in the treatment of enterococcal infections, were also favorable. Namely, the MICs of teicoplanin against *E. faecalis* were consistently reduced by over 30% regardless of the PAW treatment parameters, while both 15 and 60 min exposures caused a decrease of 25% in the MIC of vancomycin in generation 4. The summarized findings indicate a possible role of PAW applied as a support to antibiotics in the treatment of MDR infections, but the effectivity of this combinatory approach needs to be tested and verified in further experiments.

We found a significant increase in the superoxide anion concentration in *K. pneumoniae* and *E. faecalis* strains after the PAW treatments. Intracellular superoxide anion is a short-lived free radical which rapidly interacts with different biological and inorganic compounds that could potentially damage and alter the functioning of living cells [74,75,76]. Even though it is well documented that superoxide anion could have direct detrimental effects on different cellular structures [75,76,77], the complexity of oxidative stress reactions and interplay of superoxide anion with other cellular components and reactive species should not be taken out of this context. Changes in levels of total RONS were not detected. As far as the total intracellular RONS accumulation is concerned, our findings are similar to those obtained with *S. aureus* cells treated with PAW [46]. Although the findings may at least partially be related to the limitations of the testing methodologies we used [78], they are in line with the RONS present in the PAW used in our study, with the highest content of hydrogen peroxide.

For example, in the process of dismutation, hydrogen peroxide is produced from superoxide anion, which further transforms to hydroxyl radicals and is responsible for extensive damage to various biomolecules [74,75,76,77]. In addition, superoxide anion can interact with nitric oxide and form a peroxynitrite, another short-lived and highly reactive oxidant which leads to lipid peroxidation and bacterial membrane disruption [74]. The presumed importance of superoxide anion and its downstream reactive species for the antibacterial properties of PAW indicated in our study is supported in the literature, although for different bacteria samples. The role of these species was proven essential in *Escherichia coli* biofilm removal by PAW treatment [79]. A recent preprint from the same research group stressed the importance of superoxide anion in intracellular RONS accumulation, and linked the finding with 478 up-regulated and 186 down-regulated genes in the *E. coli* genome, which highlights the complexity of the cellular response to PAW exposure [80]. Rothwell et al. [43] also showed the critical relevance of superoxide against both Gram-negative *E. coli* and Gram-positive *Listeria monocytogenes*. Based on our results, the demonstrated effects of PAW on bacterial viability and susceptibility to antibiotics cannot be ascribed solely to superoxide anion and its downstream reactions, but also to oxidative stress during exposure to PAW. The cocktail of RONS both produced and accumulated in bacterial cells post-PAW exposure as well as the damaging effects of PAW-induced oxidative stress are complex and interlinked. It can only be presumed that the oxidative damage of the membrane and intracellular biomolecules resulted in either a loss of viability or in more subtle changes at the level of DNA and proteins, which led to changes in susceptibility to antibiotics [22,42,46,66]. However, more detailed mechanistic studies to understand the effects observed, particularly how PAW specifically alters susceptibility to antibiotics, are needed.

There are several limitations to the present study. Firstly, only laboratory-adapted reference strains that may not represent the full ecological and functional diversity of the species were included. Nevertheless, the presumed genetic differences between reference *K. pneumoniae* and *E. faecalis* strains and their clinical and environmental counterparts are of limited relevance in studies like ours, which are not focused on properties such as genomic diversity or virulence. Secondly, only two species of MDR bacteria were included as representatives of Gram-positive and Gram-negative bacteria, which limits the generalizability of the findings. Most of the other relevant Gram-positive and Gram-negative MDR bacteria have already been presented in the literature related to the antibacterial properties of CAP and/or PAL. The species that we have selected for this study have not been extensively investigated as specific targets for the antimicrobial activity of PAW and, especially, related changes in antibiotic sensitivity. This particularly applies to *K. pneumoniae*, which gave us an added impetus to include this species in our study. Thirdly, to obtain a deeper understanding of the processes involved in the antibacterial efficacy of PAW, the panel of testing procedures used for the detection of intracellular RONS needs to be expanded.

## 5. Conclusions

This study demonstrated, on the one hand, the inactivation activity of PAW against the superbug *K. pneumoniae,* and, on the other hand, the need for further optimization of water activation and treatment settings in order to achieve the inactivation of the Gram-positive bacterium *E. faecalis*. We investigated the effects of PAW treatments on bacterial susceptibility to traditional antibiotics, as an underexplored aspect of the activity of this emerging biocidal agent. The PAW-induced decreases in the MIC values of critical antibiotics, such as colistin against *K. pneumoniae*, and glycopeptides against *E. faecalis*, were observed. The overall findings indicate that PAW’s inactivating efficacy coupled with its capacity for the potentiation of antibiotic effects is a promising combination against MDR bacteria, while also underscoring the importance of further research into the exact mechanisms and reaction pathways that influence the bacterial response to PAW. The optimization of PAW properties, as one of the next steps in the research, should be performed in the context of particular bacterial species, represented by clinical and environmental isolates in addition to the reference laboratory strains.

## Data Availability

The additional data for this study are available from the corresponding authors upon request.

## Abbreviations

The following abbreviations are used in this manuscript:

PAW	Plasma-activated water
MDR	Multidrug-resistant
MICs	Minimum inhibitory concentration
AMR	Antimicrobial resistance
WHO	World Health Organization
CAP	Cold atmospheric plasma
RONS	Reactive oxygen and nitrogen species
ORP	Oxidation reduction potential
PALs	Plasma-activated liquids
HAIs	Healthcare-associated infections
APPJ	Atmospheric pressure plasma jet
slm	Standard liters per minute
ATCC	American Type Culture Collection
TSA	Tryptic soy agar
TSB	Tryptic soy broth
LB	Luria Bertani
CFU	Colony forming unit
MH	Mueller Hinton
DCF	Dichlorodihydrofluorescein
SPSS	Statistical Package for the Social Sciences
EUCAST	European Committee on Antimicrobial Susceptibility Testing
MRSA	Methicillin-resistant *S. aureus*
